# Associations Between Neighborhood Crime and Physical Activity Among Pregnant Women in South Carolina

**DOI:** 10.5888/pcd20.220239

**Published:** 2023-04-20

**Authors:** Kelsey R. Day, Sara Wilcox, Jihong Liu, Whitney E. Zahnd

**Affiliations:** 1 Prevention Research Center, Arnold School of Public Health, University of South Carolina, Columbia; 2 Department of Exercise Science, Arnold School of Public Health, University of South Carolina, Columbia; 3 Department of Epidemiology and Biostatistics, Arnold School of Public Health, University of South Carolina, Columbia; 4 Department of Health Management and Policy, College of Public Health, University of Iowa, Iowa City

**Figure:**
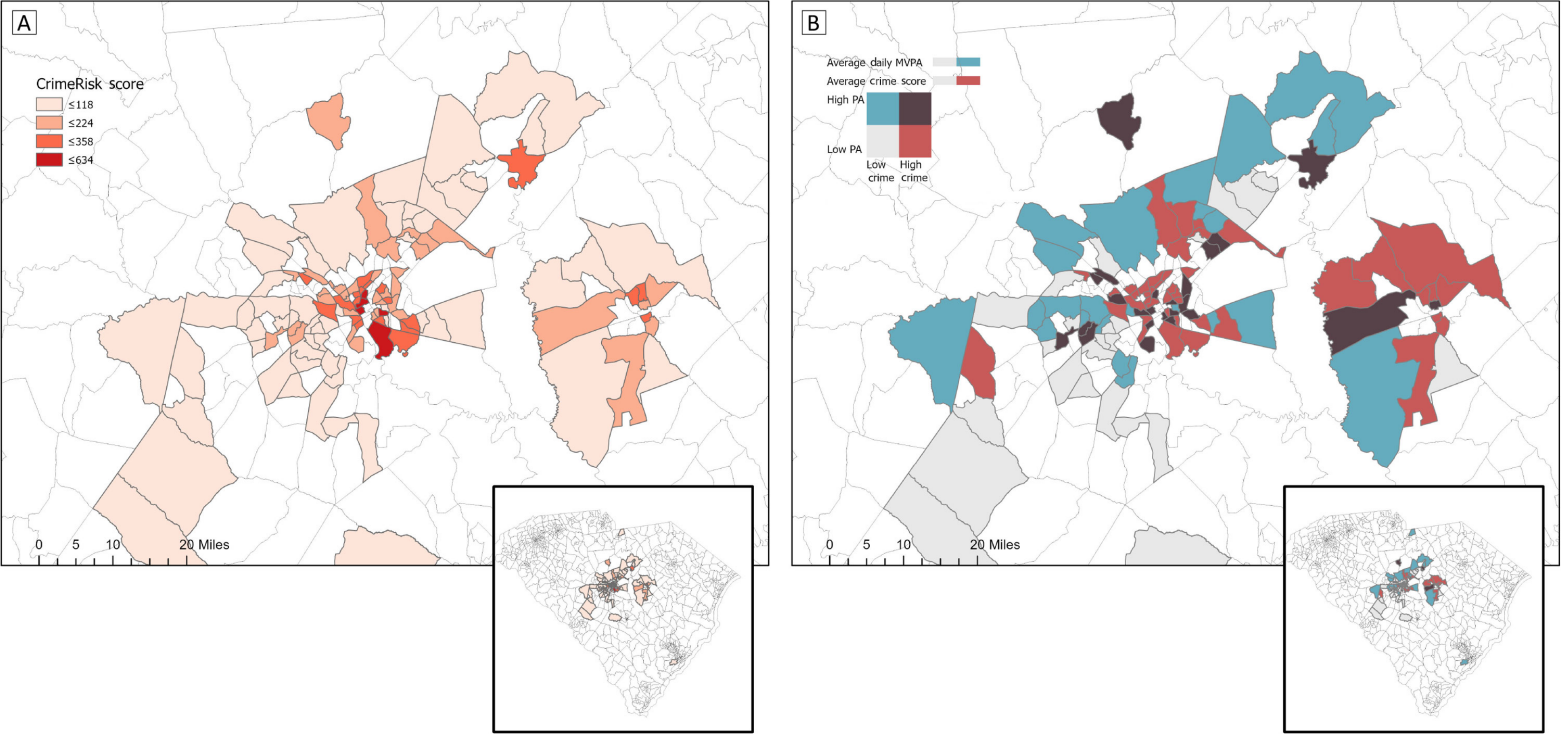
Map A shows the CrimeRisk score for the census tracts of study participants and Map B shows the bivariate relationship between MVPA and CrimeRisk score of study participants in the Health in Pregnancy and Postpartum Study, Richland, Lexington, and Sumter counties, South Carolina. Higher CrimeRisk scores indicate higher rates of crime (national mean score, 100). Lines indicate census tract boundaries. Abbreviations: MVPA, moderate to vigorous physical activity; PA, physical activity.

## Background

Regular physical activity (PA) has numerous health benefits for women before, during, and after pregnancy (1). PA reduces the risk of adverse pregnancy outcomes, such as preterm delivery and gestational diabetes (1), and mothers’ PA during pregnancy can protect their offspring against chronic disease susceptibility (2). Despite these benefits, most women do not report participating in PA during pregnancy (1). Physical changes caused by pregnancy may disrupt normal health behaviors and adversely affect PA levels (3). The built environment may also play a role in determining a woman’s ability to be active during pregnancy and the postpartum period. Neighborhood features like walkability and access to parks enhance PA and reduce the likelihood of preterm birth (4,5). In contrast, neighborhood disturbances, such as housing damage and crime, are associated with lower PA during pregnancy and adverse pregnancy outcomes (6,7). However, much of the available literature has relied on self-reported PA data, and few studies have examined the association between neighborhood safety and objectively measured PA during pregnancy. Therefore, this study aims to investigate associations between neighborhood crime and objectively measured PA among pregnant women enrolled in a randomized controlled trial.

The purpose of this study was twofold: 1) to compare pregnant women’s perceptions of neighborhood crime with their objectively measured PA and 2) to examine associations between an objective indicator of neighborhood crime and objectively measured PA. We hypothesized that safer perceived and objectively measured neighborhood environments would be associated with more steps per day and minutes per week of moderate to vigorous PA (MVPA).

## Data and Methods

Data for this study were from the Health in Pregnancy and Postpartum (HIPP) study, which has been described in detail elsewhere (8). Briefly, HIPP was a randomized controlled trial of a PA and diet intervention to prevent excess gestational weight gain among women living in South Carolina. We used data from the HIPP baseline survey that were collected between January 2015 and December 2019, including demographic information, objectively measured PA, and perceived neighborhood environment.

### Measures

At baseline, steps per day and minutes per week of MVPA were measured by SenseWear armbands (IDSA), which have been validated for use during pregnancy (9), worn continuously for 5 to 8 days (≥21 h/d, including at least 1 weekend day). Average daily MVPA was calculated for each participant.

To assess perceived neighborhood crime, participants completed a modified 11-item version of the validated Physical Activity Neighborhood Environment Scale (PANES) (10). PANES contains a single item of overall perceived crime and 2 items averaged to create an overall neighborhood crime score: “The crime rate in my neighborhood makes it unsafe to go on walks (during the day/at night).” Ratings ranged from 1, strongly disagree to 4, strongly agree. These items were reverse scored so that higher scores indicate lower perceived crime.

An objective crime score was derived for each participant’s home address at the census tract level using Esri Applied Geographic Solutions’ CrimeRisk index score (11). CrimeRisk is a standardized index for the relative risk of personal and property crimes that is calculated using historic (2014–2020) Federal Bureau of Investigation and local police data. Relative risk scores are available at the census tract level and higher; the mean national CrimeRisk score is 100 (higher score = higher crime risk).

### Spatial analysis

To derive participants’ census tract, home addresses provided at baseline were geocoded using the Census Bureau geocoder. CrimeRisk score was then added for each census tract, and descriptive analyses were performed using ArcGIS Pro (Esri) (12). Choropleth maps depicting participant location and CrimeRisk score were created, as was a bivariate map depicting mean daily MVPA and CrimeRisk score. Manual intervals were entered to create high and low classes of MVPA (high, ≥30 min/d) and CrimeRisk score (high, ≥100).

### Statistical analysis

Pearson correlations and generalized linear models adjusted for race (used as a social construct) and income (variables likely related to the dependent variable) were used to examine associations between PANES crime scores and steps per day or minutes per day of MVPA. The same analyses were then performed with CrimeRisk score.

## Highlights

Participants (N = 205) were White (56%) and African American (44%) women aged 18 to 44 years with a mean body mass index of 33.6 kg/m^2^ and a mean gestational age of 12.4 weeks. Mean steps per day was 5,360 steps (range, 1,048–14,201 steps), and mean minutes of MVPA per day was 37 minutes (range, 1–116 min). Most participants lived in Richland, Lexington, or Sumter County, South Carolina.

CrimeRisk scores ranged from 20 to 634; scores were generally higher in census tracts surrounding metropolitan centers. CrimeRisk score was also correlated with overall perceived crime (*r* = 0.43), as well as daytime (*r* = 0.45) and nighttime (*r* = 0.28) walking safety (*P* <.001).

In the adjusted linear models, MVPA was associated with both overall perceived crime score (β = 5.52, *P* = .03) and perceived daytime walking safety (β = 6.99, *P* = .02). Daytime walking safety was also associated with steps per day in the adjusted model (β = 833.86, *P* = .003). CrimeRisk score was not associated with MVPA or steps per day after adjusting for income and race (Table).

**Table:** Association Between Objectively Measured Physical Activity With Perceived and Objective Measures of Neighborhood Crime in the HIPP Trial^a^

Outcome variable	Independent variable	β	SE	*t* value	*P* value
**Perceived measure**					
Steps/d	PANES overall crime score^b^	355.24	251.37	1.41	.16
Steps/d	PANES daytime walking safety	833.86	280.34	2.97	.003
MVPA min/d	PANES overall crime score^b^	5.52	2.58	2.14	.03
MVPA min/d	PANES daytime walking safety	6.99	2.92	2.40	.02
**Objective measure**					
Steps/d	CrimeRisk score	2.03	1.43	1.42	.15
MVPA min/d	CrimeRisk score	0.02	0.01	1.30	.19

Abbreviations: HIPP, Health in Pregnancy and Postpartum study; MVPA, moderate to vigorous physical activity; PANES, Physical Activity Neighborhood Environment Scale.

a All models adjusted for income and race.

b Reverse scored so that higher scores indicate lower crime; possible range, 1 to 4; study sample mean score, 3.6 (SD, 0.62).

## Action

In this study, objectively measured neighborhood crime was not related to objectively measured PA among pregnant women. Perceived crime and safety (particularly daytime walking safety) did influence participant PA, which may be because perceptions of safety are more influential determinants of PA behavior for pregnant women (13). We found that PA among pregnant women in Richland County, South Carolina, tended to be high in downtown areas with higher crime. Therefore, policies to improve neighborhood safety should consider individual perceptions of what constitutes a safe space for PA. Policy makers could also consider examining whether such high PA and high crime neighborhoods contain environmental features that may enhance the sense of safety and, in turn, promote PA (such as street and sidewalk lighting).

Although CrimeRisk score was the best available measure of crime for this analysis, it may not be the best proxy for objective crime in the study area because it does not weight violent and nonviolent crime differently. Similar studies of objective crime and health outcomes among pregnant women have used local police data (14), which allows for analysis of different crime types. However, local police data are often difficult to obtain (15) and were not available for this study. Evidence also exists that local police data are subject to racial bias (15). Therefore, future studies should consider using CrimeRisk score to examine associations between crime and pregnant women’s PA in larger and more diverse study areas.
